# Long-term environmental enrichment affects microglial morphology in middle age mice

**DOI:** 10.18632/aging.101923

**Published:** 2019-04-29

**Authors:** Seemaab Ali, Xianglan Liu, Nicholas J. Queen, Ripal S. Patel, Ryan K. Wilkins, Xiaokui Mo, Lei Cao

**Affiliations:** 1 Department of Cancer Biology and Genetics, College of Medicine, The Ohio State University, Columbus, OH 43210, USA; 2 The Ohio State University Comprehensive Cancer Center, Columbus, OH 43210, USA; 3 Department of Biomedical Informatics, College of Medicine, The Ohio State University, Columbus, OH 43210, USA

**Keywords:** environmental enrichment, aging, amygdala, hippocampus, hypothalamus, microglia, morphology

## Abstract

Aging is associated with increased central nervous system inflammation, in large part due to dysfunctional microglia. Environmental enrichment (EE) provides a model for studying the dynamics of lifestyle factors in the development of age-related neuroinflammation and microglial dysfunction. EE results in improvements in learning and memory, metabolism, and mental health in a variety of animal models. We recently reported that implementing EE in middle age promotes healthy aging. In the present study, we investigated whether EE influences microglial morphology, and whether EE is associated with changes in expression of microglial and neuroinflammatory markers. Inflammatory cytokines and MHC-II were reduced following 12-month EE in 10-month-old mice. Long-term EE for 7.5 months resulted in broad increases in Iba1 expression in hippocampus, hypothalamus, and amygdala detected by immunohistochemistry. Quantification of microglial morphology reveal both hypertrophy and ramification in these three brain regions, without increases in microglial cell density. These data indicate that long-term EE implemented in middle age results in a microglial state distinct from that of normal aging in standard laboratory housing, in specific brain regions, associated with reduced neuroinflammatory markers and improvement of systemic metabolism.

## Introduction

Normal aging is associated with broad, systemic physiological decline. Age-related deterioration of the central nervous system (CNS) coincides with peripheral organ dysfunction, and communication between the CNS and the periphery is essential for overall homeostasis [1,2]. In this context, environmental enrichment (EE) provides a model for studying the interaction between lifestyle factors and aging [3,4]. EE is a housing condition that provides animals with novelty and complexity in their environment, from cognitive, sensory, social, and physical stimuli, and which boosts mental health [5]. Our work on EE has revealed a novel anticancer and anti-obesity phenotype in young mice mediated by a brain-fat axis: the hypothalamic-sympathoneural-adipocyte (HSA) axis. The stimuli provided by EE induces brain-derived neurotrophic factor (BDNF) in the hypothalamus, which in turn elevates the sympathetic tone to adipose tissue. Adipose tissue remodeling, including induction of beige cells and the suppression of leptin, leads to an anti-obesity and anticancer phenotype [6–8]. Our lab and others have applied EE to several solid tumor models including melanoma, colon, breast, pancreatic cancers, and glioma [6,9–12]. Moreover, we demonstrate that implementing EE after middle age promotes healthy aging characterized as decreased adiposity, enhanced glycemic control, reduced aging-associated hepatic steatosis, improved motor abilities, and inhibited anxiety [13]. The anti-aging effects of EE was not accounted for by voluntary running, but could be largely reproduced by hypothalamic overexpression of BDNF via an autoregulatory AAV-BDNF vector [14].

Microglial cells are the resident immune cells of the CNS. In their baseline state without CNS insult, these are highly ramified cells that continuously survey their territory using thin processes with multiple branches [15,16]. In this surveilling state, microglia monitor for CNS injury while also contributing to synaptic formation, function, and removal to facilitate plasticity – partly through the expression of BDNF [17,18]. As immune cells microglia can be “activated” to undergo phenotypic and morphological changes when homeostatic disturbance is detected. Sensing of immunogenic stimuli or tissue injury activate microglia to produce proinflammatory mediators to propagate an immune signal, phagocytose cellular debris, and present antigens to the immune system [19]. These “reactive” cells acquire a bushy morphology with short, retracted processes and cellular hypertrophy, with reduced surveilling process motility [20]. Moreover, old age is associated with dysfunctional microglia [21–23]. Microglia from aged animals are hyper-reactive to inflammatory stimuli, with higher basal levels of cytokine expression and exaggerated responses to activation [24]. Aged microglia exhibit shortening of processes, slower process movement, and enlarged soma volumes [25]. Functional decline of microglia is hypothesized to be progressive over the lifespan, due to accumulation of deleterious changes to these cells like increased oxidative DNA damage and accumulation of non-degradable protein and lipid aggregates. In particular, myelin breakdown in the CNS over time results in lipofuscin accumulation in microglia [26]. This inflamed and dysfunctional microglial profile of old age plays a role in the development of age-associated neurodegenerative diseases [27,28].

Many studies have also demonstrated the association of regional microglia inflammatory responses with metabolic and behavioral changes in mice. Microglial inflammatory signaling in the hypothalamus orchestrate the body’s response to high fat diet, including fat accumulation and peripheral immune cell recruitment [29]. After repeated social defeat, microglial reactivity is increased in the medial amygdala, associated with fear and anxiety-like behavior [30]. Our previous studies demonstrate that EE downregulated the expression of a cluster of cytokines and inflammatory markers in the hypothalamus of obese mice (young and old) or old mice of normal weight [11,13], including the cytokine interleukin 1 β (IL-1β) and molecules in the nuclear factor kappa-light-chain-enhancer of activated B cells (NFκB) signaling pathway, which is a key pro-inflammatory pathway of hypothalamic microinflammation [21,31]. Similar hypothalamic gene expression profiles were found in mice overexpressing BDNF in the hypothalamus [14]. These findings led us to ask whether EE could be affecting microglial cells in old age, particularly in brain regions which are involved with the response of old mice to EE. In this study, we characterized the morphological changes in the hypothalamus, amygdala, and hippocampus in middle age mice after long-term EE of 7.5 months.

## RESULTS

### 12-month EE reduces inflammation-related gene signatures in hypothalamus and amygdale

**Figure 1:**
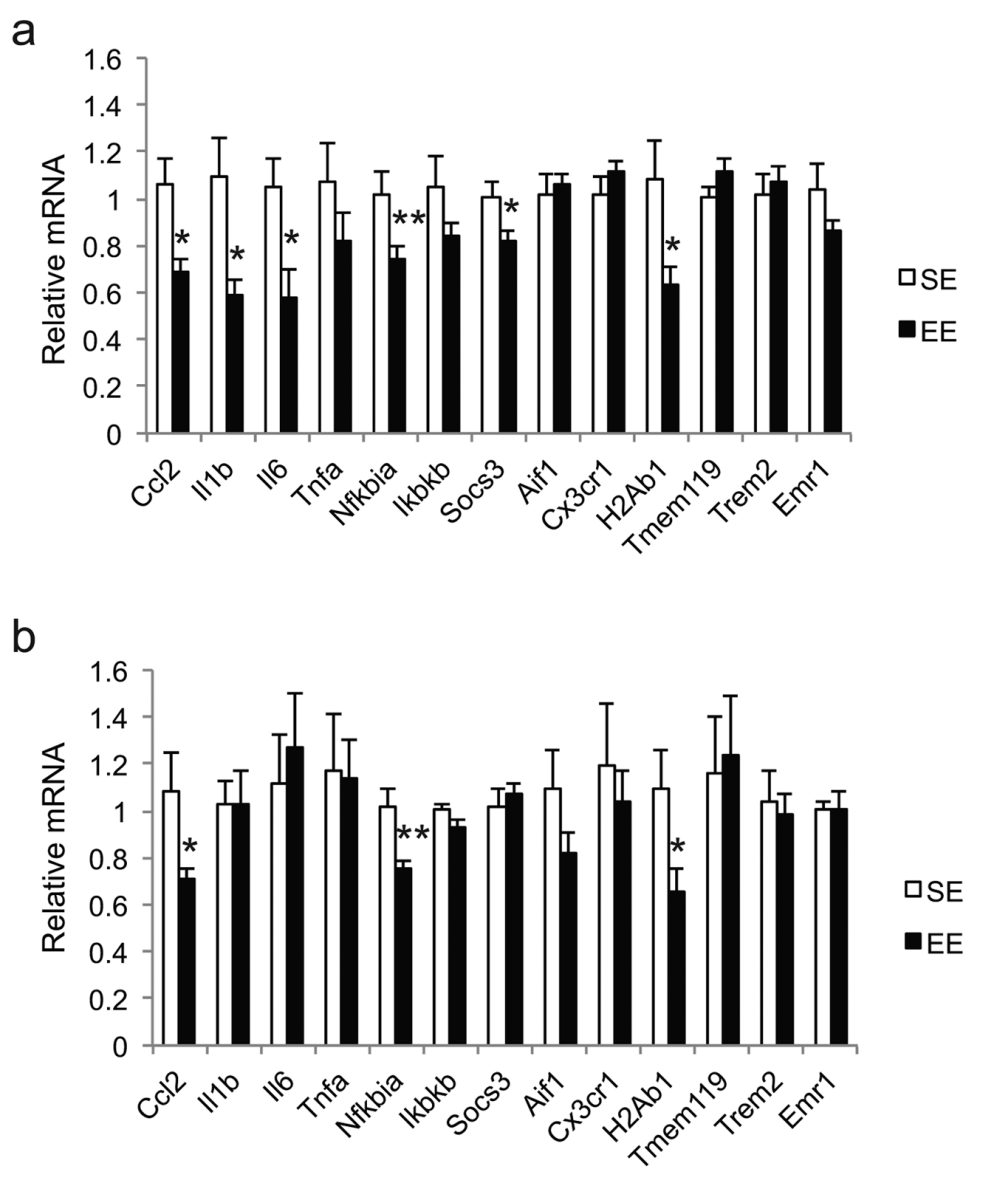
**Gene expression profiles after 12-month EE initiated at 10-month of age.** (**a**) Hypothalamus. (**b**) Amygdala. *n*=8 per group. *p<0.05, **p<0.01. Values are means ± SEM.

Previously, we reported that a cluster of inflammation-related genes was downregulated in the hypothalamus and the amygdala of 22-month old female mice after 12-month EE: Ccl2, Il1b, Il6, Nfkbia, and Socs3 [13]. In the hypothalamus the cytokines Ccl2 (encoding monocyte chemoattractant protein-1 MCP-1), Il1b (encoding IL-1β), and Il6 (encoding interleukin-6) were all downregulated following long-term EE (Figure 1a). Nfkbia (encoding NFκB inhibitor α), and Socs3 (encoding suppressor cytokine signaling 3) also showed lower expression. In the amygdala, we saw reductions in Ccl2 and Nfkbia without any change in Il1b, Il6 or Socs3 (Figure 1b). By further analysis of the previous 12-month EE study samples, we found neither region showed significant changes in Tnfa (encoding tumor necrosis factor α) or Ikbkb (encoding inhibitor of NFκB kinase subunit β).

Microglia are a primary source of cytokines in the CNS. To test whether microglia were grossly changing in response to EE, we returned to the tissue from the previous 12-month EE study to investigate a group of genes associated with microglia and their function: Aif1 (encoding allograft inflammatory factor 1, also known as ionized calcium-binding adapter molecule 1 or Iba1), Tmem119 (encoding transmembrane protein 119), Cx3Cr1 (encoding C-X3-C motif chemokine receptor 1), H2Ab1 (encoding histocompatibility 2, class II antigen A, β1, or MHC-II), Trem2 (encoding triggering receptor expressed on myeloid cells 2), and Emr1 (encoding EGF-like module-containing mucin-like hormone receptor-like 1, also known as F4/80) [32]. EE significantly reduced H2Ab1 expression in both the hypothalamus and the amygdala, while EE exhibited little effects on the transcription of other genes (Figure 1). Chronic expression of MHC-II is associated with brain aging. Approximately 25% of microglia in mice at 18-20 months of age are MHC-II positive, compared to only <3% of MHC-II positive microglia in mice at 3-4 months of age [33,34].

### EE improves glucose tolerance and body fat composition

**Figure 2:**
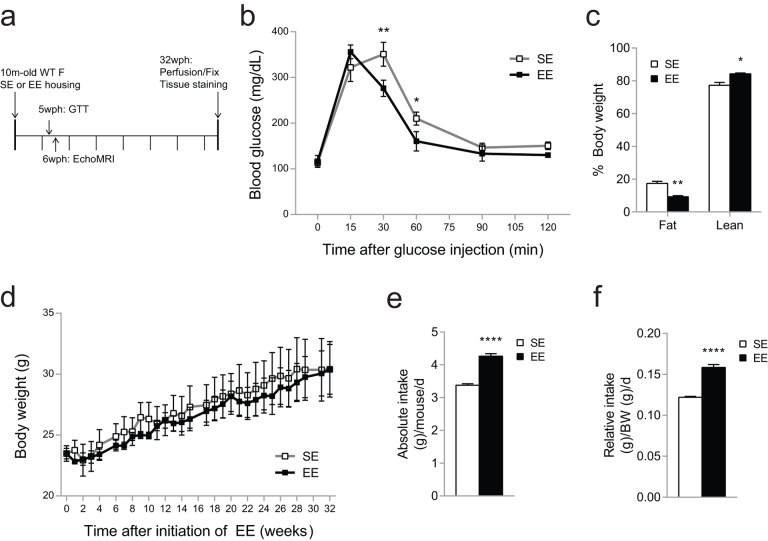
**Metabolic effects of EE in 10-month old mice during 7.5-month EE.** (**a**) Timeline of housing and studies. (**b**) Glucose tolerance test performed at 5-weeks EE. (**c**) Body composition at 6-weeks EE. (**d**) Absolute (left) and relative (right) food intake monitored for 24 weeks. (**e**) Body weight. *n*=5 per group. *p<0.05, **p<0.01, ***p<0.001, ****p<0.0001. Values are means ± SEM.

This evidence suggested there may be a phenotypic difference in microglia between the housing conditions. The aged microglial phenotype is thought to be a result of an accumulation of deleterious cellular changes over time. We hypothesized that, if the rate of this accumulation was different between housing conditions, the difference in microglial phenotype after EE would be detectable at an age when microglia otherwise show age-related changes. Therefore, we housed 10-month old mice in either SE or EE for 32 weeks, or 7.5 months, when mice are entering early old age (Figure 2a) [35]. Environmental (EE) or genetic (hypothalamic overexpression of BDNF) activation of the HSA axis is associated with metabolic improvement including reduced adiposity and improved glycemic control in middle age mice [13,14]. In order to detect early efficacy of EE, we assessed adiposity and glycemic control at five weeks post-housing. Five weeks of EE housing improved glucose tolerance (Figure 2b). EchoMRI revealed significantly reduced fat mass and increased lean mass in EE mice at 6 weeks (Figure 2c). There was no significant body weight difference between housing conditions across the 7.5-month period (Figure 2e) while EE mice consumed more food (Figure 2d). Overall, we observed the metabolic outcomes induced by EE consistent with previous studies [13].

### Microglia are hypertrophied and ramified after long-term EE

**Figure 3:**
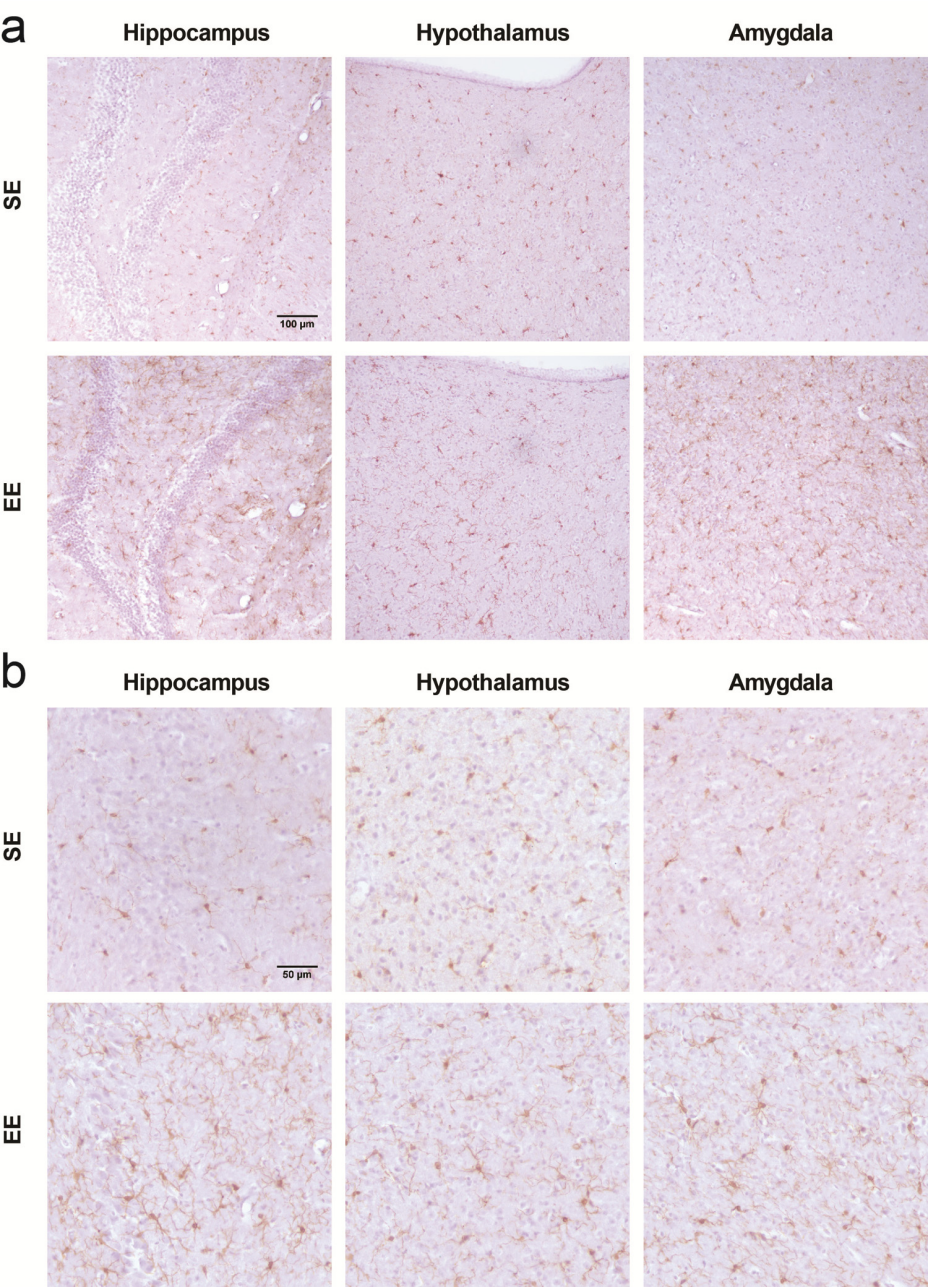
**Microglial changes observed after 7.5-month EE.** (**a**) Representative fields of Iba1 stained IHC in the hippocampus (left), hypothalamus (middle), and amygdala (right) at 20x magnification. (**b**) 40x magnification. Scalebars, (**a**) 100 µm and (**b**) 50 µm.

We next examined Iba1 IHC staining across the brain to investigate microglial changes following long-term EE of 7.5 months. Broadly, Iba1 staining appeared prominent in the hippocampus (HA), hypothalamus (HYP), and amygdala (AMY) of EE mice relative to SE mice (Figure 3). High magnification survey of these regions revealed SE microglia to have fewer and shorter observable processes in mostly non-reactive morphologies, consistent with previous reports on microglial morphology in older mice. In contrast, microglia from EE mice showed highly ramified morphology in all of the three brain regions, with variability across subfields and nuclei. This ramified morphology from EE mice was similar to young adult microglia, as seen in 3 month old female mice (Supplementary Figure 1). Unlike Iba1, GFAP showed no gross differences in astrocytic reactivity or morphology (Supplementary Figure 2).

**Figure 4:**
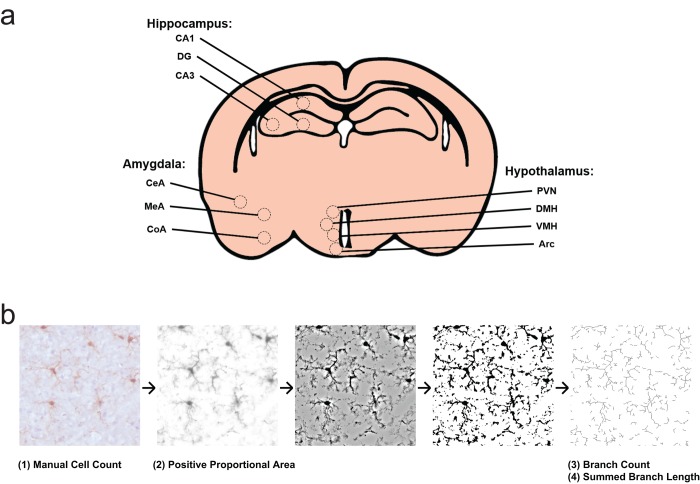
**Experimental design for morphology survey.** (**a**) Select subfields and nuclei analyzed within the hippocampus, hypothalamus, and amygdala. CA1, cornu ammonis 1; CA3, cornu ammonis 3; DG, dentate gyrus; Arc, arcuate nucleus; DMH, dorsomedial hypothalamic nucleus; PVN, paraventricular nucleus; VMH, ventromedial hypothalamic nucleus; CeA, central nucleus of the amygdala; CoA, cortical nucleus of the amygdala; MeA, medial nucleus of the amygdala. (**b**) Representation of photomicrograph processing for morphology survey. Cells are counted from original images by blinded scorers. Images are then deconvoluted in order to visualize only DAB positive area, then thresholded to binary images at a predetermined intensity to measure Iba1 proportional area. Deconvoluted images are filtered and again made binary, in order to skeletonize cellular processes. The Analyze Skeleton plugin is then used to identify and measure number of branches and branch length.

Based on these observations, we quantified the microglial cell morphology changes of prominent nuclei within HA, HYP, and AMY (Figure 4a). For subsequent analyses, we separated each region by subfield or nucleus: the hippocampus was divided into cornu ammonis 1 (CA1), cornu ammonis 3 (CA3), and dentate gyrus (DG); the hypothalamus was divided into arcuate nucleus (Arc), dorsomedial hypothalamic nucleus (DMH), paraventricular nucleus (PVN), and ventromedial hypothalamic nucleus (VMH); and the amygdala was divided into central nucleus of the amygdala (CeA), cortical nucleus of the amygdala (CoA), and medial nucleus of the amygdala (MeA). Microglial features were extracted at the level of field photomicrographs (Figure 4b).

**Figure 5:**
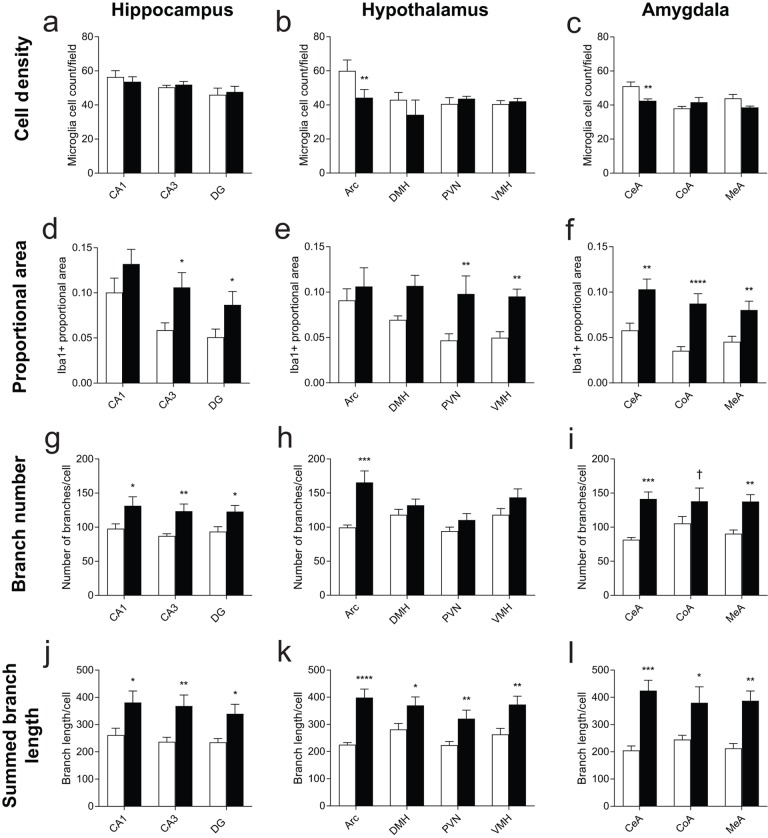
**Morphology measurements across hippocampus, hypothalamus, and amygdala nuclei.** (**a**-**c**) Cell density measures from nuclei within the (**a**) hippocampus, (**b**) hypothalamus, and (**c**) amygdala from fields of approximately 0.187 mm^2^. In (**b**), Arc is adjusted to represent the density of cells in Arc given an equivalent field area. **(d**-**f**) Iba1 positive-staining proportional area. (**g**-**i**) Skeleton branches counted from fields of each nucleus. (**j**-**l**) Total branch length from fields of each nucleus. *n*=4 for SE, 5 for EE. *p<0.05, **p<0.01, ***p<0.001, ****p<0.0001, †*p*<0.06. Values are means ± SEM. For statistical summary, see Supplementary Table S1.

Cell density was roughly uniform within each of the three brain regions analyzed (Figure 5a-c). Cell decreases were observed only in both Arc and CeA after EE (Figure 5b, c). Iba1 positive proportional area was observed higher across all sections of EE compared to those of SE (Figure 5d-f). Iba1 positive area measurements are sensitive to changes in microglia cell number as well as microglial morphology [36]. Without increases in cell density, Iba1 positive area increases point to cellular hypertrophy. HA Iba1 increases were seen within subfields CA3 and DG (Figure 5d). Within HYP, proportional area increases were restricted to PVN and VMH. No differences were found in Arc, in which we also observed decreased cell density (Figure 5e). AMY exhibited significant proportional area increases across all three nuclei in EE, with the greatest increase in CoA (Figure 5f).

We then directly measured microglial ramification by analyzing microglial skeletons for branch number and branch length, normalized by the number of microglia identified in each field [37]. EE housing resulted in more numerous and longer microglial process in each brain region (Figure 5g-l). Arc, but not other HYP nuclei, shows increased microglial branch counts (Figure 5h). Branch count increases were also observed for CeA and MeA, and trended towards significance in CoA (Figure 5i). All other nuclei showed consistent, significant increases in both the number of microglial processes and the total length of measured microglial processes after long-term EE compared to SE, suggesting a robust difference in microglial branching phenotype across all three brain regions.

## DISCUSSION

Microglia, as the main neuroimmune cells, exert three essential functions: sensing, housekeeping, and defense under physiological and pathological conditions [32]. Microglia can take on several different activation states in order to accomplish these functions, from pathogen-responsive and inflammatory states to tissue repair, regulatory, or homeostatic phenotypes [38]. Microglia are sensitive to environmental perturbations [39]. Stressors which induce anxiety-like behavior in mice are also associated with increased microglial Iba1 immunoreactivity across the hippocampus, hypothalamus, and amygdala [40]. Previously, we identified long-term EE produces a strong anxiolytic effect in aged mice [13]. Intriguingly, here we identify that EE is also associated with increased Iba1 immunoreactivity.

EE has also been shown to significantly alter microglia in a variety of healthy and pathological contexts [41–46]. Most studies focus on hippocampal microglia [41,42,44–47], and implicate hippocampal microglia in mediating EE-induced neuroplasticity, such as increased dendritic arbors, neurogenesis, and improvements to learning and memory [45–48]. These studies suggest EE significantly alters microglial functional state, and that microglial morphology changes correspond with changes in activity or activation state in these cells.

By contrast with post-EE microglia, inflammatory microglia as present in aged animals actively inhibit hippocampal neurogenesis and long-term potentiation [49]. Typically, aged microglial morphologies are described as deramified compared to young brain microglia [50]. Hefendehl and colleagues quantified fine primary filaments from cortical microglia, concluding aging results in shorter microglial processes without decreasing the number of processes per cell [25]. CA3 microglia also exhibit reduced surface area, branch volume, and branch length in aged mice [51]. These aged morphologies are associated with increased pro-inflammatory and anti-inflammatory cytokine production [52].

Our results demonstrate a morphological change occurring in microglia in aged mice after EE. We identified parameters that could be assessed with IHC that were meaningful with respect to the function of microglia in the brain regions tested. Microglial cell counts show that proliferation or infiltration was not occurring in response to EE, and that microglial cell count was decreasing in two nuclei important for behaviors altered by long-term EE: feeding behavior (Arc) and fear response (CeA). Iba1 immunoreactivity assessed by percent area show that hypertrophy is occurring in the absence of cellular proliferation or infiltration. Finally, our branching metrics suggest that this is not the normal hypertrophy seen in aged microglia associated with inflammatory responses; rather, we see increased branch number and branch length per microglia across several brain regions. Overall, compared to aged morphologies from SE housing, long-term EE initiated at middle age led to hypertrophied microglia, as measured by Iba1 area, with longer and more numerous branches in nuclei of the hippocampus, hypothalamus, and amygdala. This is consistent with a previous report that long-term EE of female albino Swiss mice for 16-20 months resulted in increased microglial branch number and branch length in CA3, compared to SE housing [51]. Insofar as these morphology results can be associated with functional states of microglia, we also observed changes in gene expression in both the hypothalamus and the amygdala after long-term EE. MHC-II expression was decreased in both the hypothalamus and amygdala after long-term EE. This study is limited to morphological analysis and profiling of a small set of selected genes. Future studies are required to assess microglial functional status.

Relative to the hippocampus, information on hypothalamic and amygdalar microglial response to EE is sparse. We are particularly interested in the hypothalamus as a key CNS regulator of energy balance, immune function, and stress. Our studies in various murine models have demonstrated that EE alters the body’s three major adaptive systems—nervous, endocrine, and immune systems. Hypothalamic BDNF orchestrates multiple processes along each system, providing an important target for manipulation with therapeutic potential. Recently we have identified broad metabolic improvements in middle age mice after EE, including reduced adiposity and improved glucose tolerance as well as prevention of aging-related liver steatosis (Figure 2) [13]. The present study design does not account for individual variability in metabolic response to EE or the steady-state maintenance of metabolic improvements over time. We previously identified adipose tissue reductions and glucose tolerance improvements at 6 weeks, 2 months, 3 months, and 32 weeks (GTT) in 10-month old mice housed in EE, relative to parallel SE mice in each study [13]. The reliability of these outcomes at several time points across several randomized mouse cohorts suggests broad applicability of EE despite individual level differences, as well as progressive or steady state improvement in metabolic outcome over long periods of time.

In addition to the metabolic changes, EE also led to downregulation of inflammatory cytokines in the hypothalamus (Figure 1a). These EE-associated changes are not accounted for by wheel running exercise alone. However, they are largely reproduced by hypothalamic gene transfer of BDNF via an autoregulatory rAAV vector [13,14]. In the present study we observed morphological changes in hypothalamic microglia after long-term EE exposure. Notably, reactive microglia in the hypothalamus, particularly in Arc, contribute to the development of obesity [29,53,54]. Our results show significantly fewer Arc microglia after EE with similar overall Iba1 coverage, suggesting these cells are also hypertrophied relative to SE (Figure 5b,e). Reducing Arc microglia may be one mechanism by which EE leads to reduced adiposity in aged mice. Investigations on the role of microglia in EE-induced metabolic modulation are currently underway.

Hypothalamic BDNF has been identified as the key mediator for EE-induced metabolic, immune, and anticancer effects [6,7,55]. The major source of BDNF in adult brain is neurons. AAV1 (or AAV2)-mediated upregulation or downregulation of BDNF reproduces or blocks EE’s effects, respectively [6,7]. Because these AAV serotypes are neurotrophic, these data are consistent with neuron-derived hypothalamic BDNF playing the major role in EE outcomes. Elsewhere in the brain, BDNF has also been implicated as a key mediator of neurogenesis and enhanced memory after EE [56]. Importantly, BDNF can be detected in microglia, as well. A recent report demonstrated that microglia-derived BDNF acts on the TrkB receptor on neurons to promote learning-dependent synapse formation [18]. The multiple roles of BDNF in EE, the functional role of BDNF from microglia in synaptic plasticity, and the impact of EE on microglia form and function [57,58], together suggest a possible crosstalk between microglia and neurons involving BDNF important for EE outcomes. Further studies are needed to tackle the interesting question whether hypothalamic BDNF mediates the EE-induced microglia morphological and/or functional alterations.

In summary, our studies demonstrate that long-term EE initiated at middle-age results in microglial morphology changes in several brain regions, primarily with increases in cellular hypertrophy and ramification. These changes correspond with improvements in metabolic and behavioral outcomes. Further characterization and manipulation of the activation state of microglia in these brain regions will provide information on the underlying mechanisms of EE and improved lifestyle in promoting healthy aging.

## MATERIALS AND METHODS

### Animals and housing

Female 10-month old WT C57BL/6N mice (National Institute on Aging, Aged Rodent Colonies) were randomized to live in either EE or SE conditions. For EE, mice were housed in large cages (63 cm x 49 cm x 44 cm, 5 mice per cage) supplemented with running wheels, tunnels, igloos, huts, retreats, wood toys, and a maze in addition to standard chow and water. For SE, mice were housed in standard mouse cage of 30.5 cm x 17 cm x 15 cm (5 mice per cage). Mice were housed in temperature (22-23 ºC) and humidity (30-70%) controlled rooms under a 12:12 light/dark cycle, with access to food and water *ad libitum*. Mice were fed with normal chow diet (NCD, 11% fat, caloric density 3.4kcal/g, Teklad (from Envigo), Indianapolis, IN). All animal experiments were carried out in compliance with the regulations of the Ohio State University Institutional Animal Care and Use Committee. In the study for mRNA expression within the hypothalamus and the amygdala, animals were housed in EE for 12 months and were sacrificed at 22 months of age (RT-PCR, *n*=8 per group) [13]. In the study for microglial morphology, animals were housed for 32 weeks until the study was ended at 17.5 months of age (*n*=4 for SE, *n*=5 for EE). While mice were housed at 5 mice per cage for the duration of the study, one mouse was excluded from our final SE set due to significant frailty and failure to gain weight. Body weight was measured weekly for the duration of the study, except weeks where noted for staff or access reasons. Food intake was measured weekly across the study for a total of 24 measurements. Food intake is reported as the remaining food weight, divided by the number of days between each measurement, divided by the number of mice per cage. Relative food intake is the same measurement, normalized to per gram body weight in each cage rather than the number of mice.

### Quantitative RT-PCR

Hypothalamus and amygdala were dissected at sacrifice. RNA was isolated using Qiagen RNeasy Mini kit with RNase-free DNase treatment (Germantown, MD), and first-strand cDNA was reverse transcribed using TaqMan Reverse Transcription Reagents (Applied Biosystms, Foster City, CA). We performed quantitative PCR on a StepOnePlus Real-Time PCR System (Applied Biosystems) with Power SYBR Green PCR Master Mix (Applied Biosystems). Data was calibrated to endogenous control Hprt1 for hypothalamus and Ppia for amygdala, and relative gene expression was quantified using the 2^-ΔΔCT^ method. Our primer sequences are available on request.

### Glucose tolerance test

After an overnight fast (>16 hr), mice were injected intraperitoneally with a glucose solution (2 g/kg body weight). Blood was obtained from the tail before injection and at 15, 30, 60, 90, and 120 min after glucose injection. Blood glucose concentrations were measured with a portable glucose meter (Bayer Contour Next, Parsippany, NJ).

### Body composition

EchoMRI was used to measure body composition of fat, lean, free water, and total water masses in live mice without anesthesia. EchoMRI imaging was performed with an EchoMRI Analyzer (EchoMRI, Houston, TX) at the Small Animal Imaging Core of The Dorothy M. Davis Heart & Lung Research Institute, Ohio State University.

### Perfusion

After 32 weeks post housing, mice were anesthetized and transcardially perfused with PBS, followed by 4% paraformaldehyde (PFA) (Sigma, St. Louis, MO) in PBS. Fixed brains were extracted and incubated in 4% PFA on a rocker overnight at 4ºC. Brains were then rinsed in PBS before being submerge in 30% sucrose in PBS with 0.03% sodium azide for at least 3 days at 4ºC.

### Immunohistochemistry

Fixed brains were sectioned into 30-µm free-floating slices on a ThermoSceintific HM525NX cryostat (Waltham, MA), and subjected to citrate-based antigen retrieval following by incubaton with rabbit anti-Iba1 (FUJIFILM Wako Chemicals USA, Cat. No.019-19741, Richmond, VA, 1:1000) overnight at 4ºC. The sections were visualized with DAB and counterstained with hematoxylin.

### Image collection

Microscopy was performed on a Nikon Eclipse 50i microscope (Nikon Instruments, Melville, NY) at 40x magnification. Photomicrographs were collected using an attached Axiocam 506 color camera (Zeiss Microscopy, Peabody, MA). For each animal, 6-8 sections randomly sampled between -0.8 mm to -2.1 mm from bregma were visualized. Nuclei from the hippocampus (CA1, CA3, DG), hypothalamus (Arc, DMH, PVN, VMH), and amygdala (CeA, CoA, MeA) were identified from these sections according to the Allen Adult Mouse Brain Atlas. From each mouse, 6-8 fields were collected of each nucleus for analysis, except for PVN, from which 2-4 fields were analyzed per mouse. In addition, datasets for Arc and DMH each include 1 mouse from which only 4 fields were identified, and VMH includes 2 mice from which only 4 fields were identified.

### Image analysis

The image analysis procedure is summarized in Figure 4b. Image manipulation and analysis was performed using the FIJI distribution of ImageJ 1.51n, release date 2017 May 30, available at *fiji.sc* [59,60]. Manual cell counts were performed by blinded scorers using the Multi-Point tool. Cell density was determined by averaging cell counts across the area of one 40x photomicrograph. Iba1 positive staining area was measured by automated Digital Image Analysis [36]. After color deconvolution to isolate the DAB stain, a predetermined positive staining threshold was used to determine the proportion of area positive for DAB staining. For Arc fields, the shape of analysis area was drawn prior to thresholding, and this area was used to normalize the Arc cell counts to the field area analyzed in other nuclei. Skeleton Analysis was conducted according to published protocols, using an automated version of the algorithm by Young & Morrison, using the Analyze Skeleton plugin of FIJI and with a single free-branch cutoff of 1.75 µm [37].

Each image was processed for each of four measurements (cell density, Iba1 positive proportional area, branch number, and summed branch length), after which results were averaged within each nucleus per mouse and statistical analyses were performed using *n*=4 for SE and *n*=5 for EE.

### Statistical analysis

Data are expressed as mean ± SEM. We used GraphPad Prism v7.00 (GraphPad, La Jolla, CA) and SPSS Statistics v25 (IBM, Armonk, NY) to analyze each data set. Before analysis, all data were tested for normality by Shapiro-Wilk test. Image data were subsequently log(2) transformed to fit normality assumptions for our analyses. Student’s *t*-tests were used to compare the difference between groups for quantitative RT-PCR ΔCt values, food intake, and body composition. Two-way repeated measures analysis of variance (ANOVA) was performed on time course measurements (body weight, GTT), using housing condition as a between-subjects factor and time as a within-subjects factor. These were followed by planned comparisons between housing conditions using Fisher’s Least Significant Difference (LSD) test. Image measurements were also examined by a two-way randomized block ANOVA, with housing condition as a between-subjects factor and nucleus as a within-subjects factor, followed by Fisher’s LSD tests. Statistical tests were not adjusted to minimize type I error from multiple comparisons.

## Supplementary Material

Supplementary Figures

Supplementary Table
