# Supplementary material for: Long-term environmental enrichment affects microglial morphology in middle age mice
Source: Aging (Albany NY). 2019 Apr 29;11(8):2388–402. doi: 10.18632/aging.101923 (PMC6519992; doi:10.18632/aging.101923)
Supplement: Supplementary Figures [file aging-11-101923-s002.pdf]

SUPPLEMENTARY FIGURES

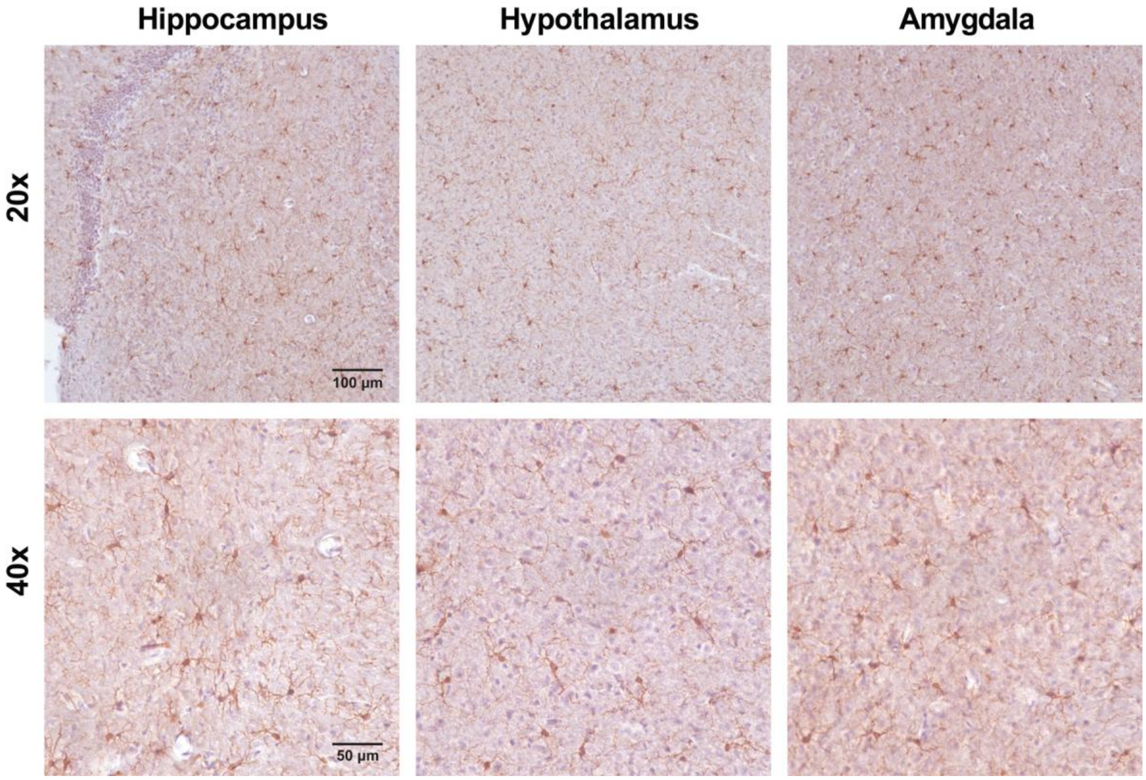

Supplementary Figure 1. Microglia observed in young adult female mice. Representative images of Iba1 stained IHC in the hippocampus (left), hypothalamus (middle), and amygdala (right) of 3 month old WT female mice at 20x (top) and 40x (bottom) magnifications. Scale bars, 100 µm (top) and 50 µm (bottom).

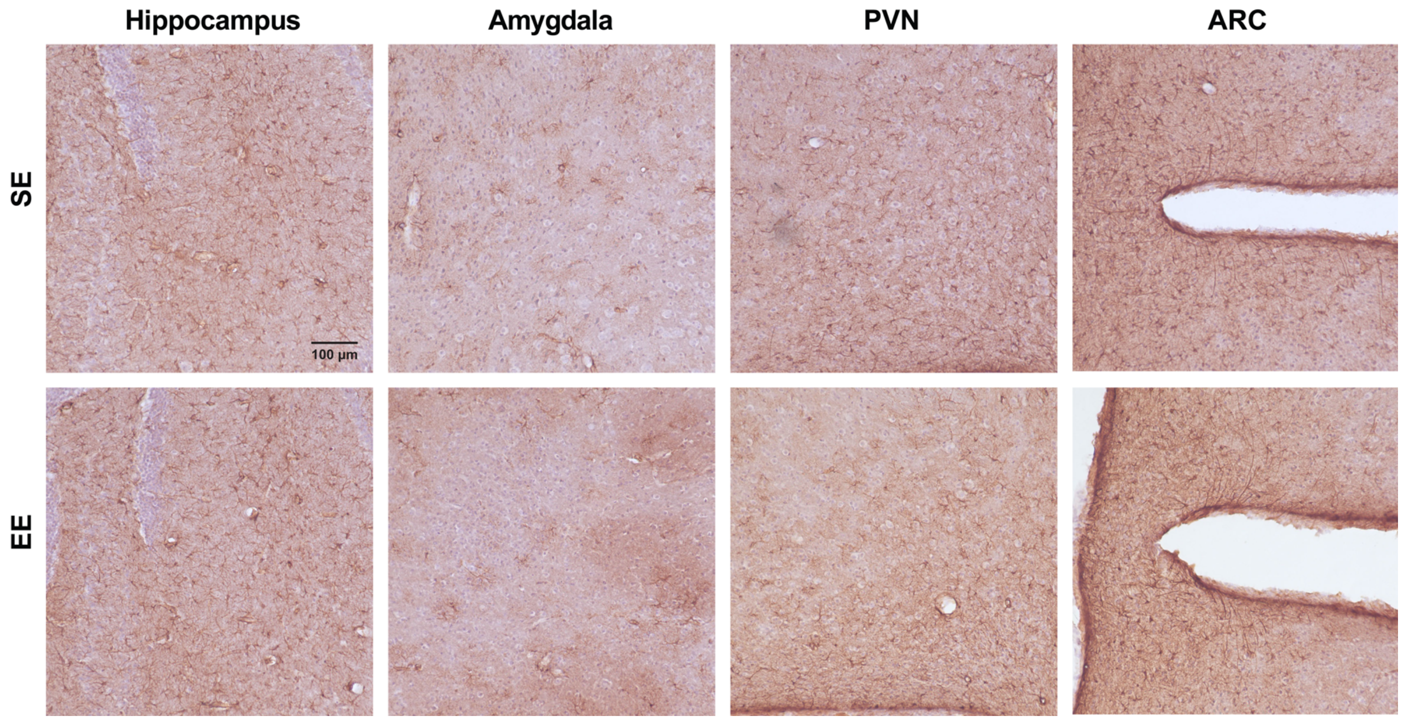

Supplementary Figure 2. Astrocytes observed after 7.5-month EE. Representative images of GFAP stained IHC in the hippocampus (left), amygdala (center left), paraventricular nucleus of the hypothalamus (center right), and arcuate nucleus of the hypothalamus (right) at 20x magnification. Scale bar, 100 µm.
